# Effect of Cold Atmospheric Plasma Jet Associated to Polyene Antifungals on *Candida albicans* Biofilms

**DOI:** 10.3390/molecules26195815

**Published:** 2021-09-25

**Authors:** Lady Daiane Pereira Leite, Maria Alcionéia Carvalho de Oliveira, Mariana Raquel da Cruz Vegian, Aline da Graça Sampaio, Thalita Mayumi Castaldelli Nishime, Konstantin Georgiev Kostov, Cristiane Yumi Koga-Ito

**Affiliations:** 1 Science Applied to Oral Health, Graduate Program of Institute of Science and Technology, São Paulo State University (Unesp), São José dos Campos 12245-000, Brazil; lady.leite@unesp.br (L.D.P.L.); mariana.vegian@unesp.br (M.R.d.C.V.); aline.sampaio@unesp.br (A.d.G.S.); 2 Oral Biopathology, Graduate Program of Institute of Science and Technology, São Paulo State University (Unesp), São José dos Campos 12245-000, Brazil; macoliveira12@gmail.com; 3 Leibniz Institute for Plasma Science and Technology, 17489 Greifswald, Germany; thalita.nishime@inp-greifswald.de; 4 Department of Physics, Guaratinguetá Faculty of Engineering, São Paulo State University (Unesp), Guaratinguetá 12516-410, Brazil; konstantin.kostov@unesp.br; 5 Department of Environment Engineering, Institute of Science and Technology, São Paulo State University (Unesp), São José dos Campos 12247-016, Brazil

**Keywords:** *Candida albicans*, antifungal, cold plasma

## Abstract

The increasing incidence of antifungal resistance represents a great challenge in the medical area and, for this reason, new therapeutic alternatives for the treatment of fungal infections are urgently required. Cold atmospheric plasma (CAP) has been proposed as a promising alternative technique for the treatment of superficial candidiasis, with inhibitory effect both in vitro and in vivo. However, little is known on the association of CAP with conventional antifungals. The aim of this study was to evaluate the effects of the association between CAP and conventional polyene antifungals on *Candida albicans* biofilms. *C. albicans* SC 5314 and a clinical isolate were used to grow 24 or 48 h biofilms, under standardized conditions. After that, the biofilms were exposed to nystatin, amphotericin B and CAP, separately or in combination. Different concentrations of the antifungals and sequences of treatment were evaluated to establish the most effective protocol. Biofilms viability after the treatments was compared to negative control. Data were compared by One-way ANOVA and post hoc Tukey (5%). The results demonstrate that 5 min exposure to CAP showed more effective antifungal effect on biofilms when compared to nystatin and amphotericin B. Additionally, it was detected that CAP showed similar (but smaller in magnitude) effects when applied in association with nystatin and amphotericin B at 40 µg/mL and 60 µg/mL. Therefore, it can be concluded that the application of CAP alone was more effective against *C. albicans* biofilms than in combination with conventional polyene antifungal agents.

## 1. Introduction

The incidence of fungal infection increased significantly in the last decades and, nowadays, is considered an important cause of mortality worldwide [1]. Most of these infections are caused by *Candida* spp. that are commensal microorganisms that can be isolated from the human oral cavity, skin, gastrointestinal and urogenital systems [2]. From the total of 200 species, approximately 20 are associated to human diseases [3]. *Candida albicans* is one of the most isolated species from the oral cavity, often associated with candidiasis [1]. In addition to virulence factors that facilitate host invasion, biofilm formation improves adherence, protects *C. albicans* cells and can guarantee resistance to the pathogen in adverse environmental conditions [2].

Under the presence of predisposing factors, *Candida* can cause infections that can vary from superficial muco-cutaneous diseases to systemic invasive processes [4]. Systemic predisposing factors include the use of wide spectrum antibiotics or immunosuppressants, transplants, use of catheters, diabetes, severe malnutrition, anti-neoplastic radio or chemotherapy and HIV infection [5,6]. In the case of oral candidiasis, other local factors can also favor the occurrence of disease, such as smoking, use of inadequate oral prosthesis, and xerostomia, among others [7].

The treatment of oral candidiasis may be done topically or systemically. The topic therapies that are used more frequently are applied directly on the lesions, and systemic ones are mostly used when disseminated infection occurs. Topic antifungals have few and mild adverse effects, since the absorption is limited and the interaction with other systemic medication is rare [8]. However, the unpleasant flavor of the medication and long treatments are limitations for the adherence to therapeutic protocol.

Topical nystatin 100,000 UI/mL and amphotericin B (50 mg) are the most frequently used treatments for oral candidiasis [9]. Nystatin is the first antifungal used for the treatment of cutaneous and mucocutaneous candidiasis and is available in topic creams and oral rinses [10,11]. Amphotericin B is also an alternative for the treatment of oral candidiasis but it is not available in many countries [8]. Its use is only suggested for topical use, but it is widely administered in systemic fungal infections [3].

Despite the presence of commercially available antifungals, the increasing occurrence of conventional antifungals resistance is also a challenge [12,13,14,15,16]. The evolution of antifungal resistance is worrisome, mainly due to the limited number of antifungal drugs available [1] and the low number of new antifungals discovered in recent decades [17]. For these reasons, the search for new alternative methods is necessary.

In this context, cold atmospheric plasma (CAP) appears as a promising alternative. Plasma is a complex mixture of gas molecules, active radicals, ions, electrons and energetic photons. Individually, each one of these reactive species is known to induce microbial inactivation. Moreover, in plasmas all these agents act synergistically, thus the antimicrobial effect is greatly enhanced. The non-thermal plasma has been investigated for several applications with potential antimicrobial effects [4,18,19]. The inhibitory effect of CAP on the modulation of adhesion and filamentation of *C. albicans* have been reported in vitro [20,21,22,23]. After 5 min exposure to helium CAP, a significant reduction in cells in *C. albicans* biofilm was also observed [21]. In vivo experiments showed inhibition of tissue hyphae invasion with the same time of exposure to CAP, which was also verified using nystatin [24]. Despite this, only fungistatic effects were verified.

Thus, although the antimicrobial properties of CAP have been explored, little is known about the effect of the synergistic interactions of CAP and antifungal agents on *C. albicans* biofilms. Synergistic interactions depend on the interaction of the components, with an increase in the activity of each one, which can allow an increase in the effectiveness of treatments with dose reduction, toxicity or deceleration of antifungal resistance [25]. The reduction in sessile minimal inhibitory concentrations (SMICs) of fluconazole, amphotericin B, and caspofungin for *Candida* biofilms after exposition to He/O_2_ (2%) plasma microjet (PMJ) was reported [26]. In a recent study, synergistic effects between CAP and conventional antifungal agents were investigated using a disc diffusion assay [22]. The authors reported a significant increase in the growth inhibition halo of *C. albicans* treated with fluconazole after previous exposure to plasma produced with He and O_2_ (2%), for 180 and 210 s, compared to the control without exposure to CAP.

Therefore, this study aimed to evaluate the effects of the association between helium CAP and the conventional antifungal agents nystatin and amphotericin B on *C. albicans* biofilms, varying CAP exposure times and antifungal concentrations in different associations, evaluating the synergistic potential of the treatments.

## 2. Results

### 2.1. Antifungals Minimal Inhibitory Concentrations

The MIC values determined for nystatin and amphotericin B were 8 µg/mL and 0.5 µg/mL, respectively, for both strains of C. albicans used in this study. Ten times these values was used for the treatment of biofilms in isolated treatments with antifungals, and as a basis for associated treatments.

### 2.2. Antibiofilm Effect of Isolated Treatments

**Figure 1:**
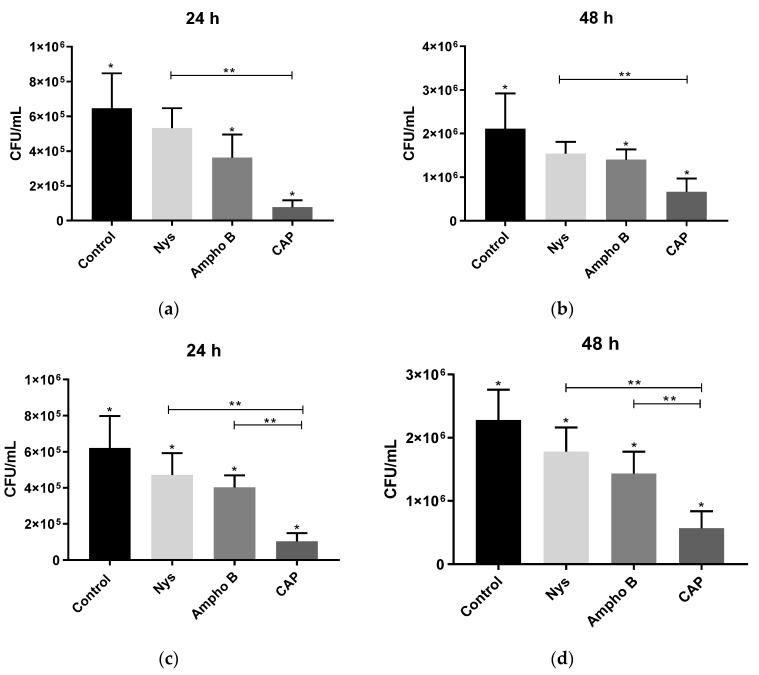
Values of biofilms viable cells after treatments with polyenes antifungals (nystatin, ampho B) and CAP, separately. The biofilms of 24 and 48 h of reference strain SC5314 (**a**,**b**) and clinical isolate P29 (**c**,**d**) were exposed with each treatment for 5 min. Control = no treatment; Nys = treatment with 80 µg/mL of nystatin; Ampho B = treatment with 5 µg/mL of amphotericin B; CAP = treatment with cold atmospheric plasma. (*) indicate significant differences (*p* < 0.05) between the groups and the control. (**) indicate significant differences (*p* < 0.05) between the treatments.

The isolated treatments consisted of the application of nystatin and amphotericin B (10 times MIC; 80 and 5 µg/mL, respectively) and CAP for 5 min. Significant reductions (*p* < 0.005) of viable cells were observed in the 24 h biofilms of both strains of C. albicans after treatments with amphotericin B and CAP, in comparison with the untreated control (Figure 1a,c). The same was observed for the 48 h biofilms (Figure 1b,d).

Regarding biofilms (24 and 48 h) formed with the clinical isolated (P29), it was still possible to observe a significant reduction (*p* < 0.05) after treatment with nystatin, compared to the control. Exposure to CAP proved to be the most efficient method, compared to conventional antifungals, with a reduction in CFU/mL in a decreased manner (*p* < 0.05) for both strains.

### 2.3. Effective Antibiofilm Conditions in Associated Treatments

**Figure 2:**
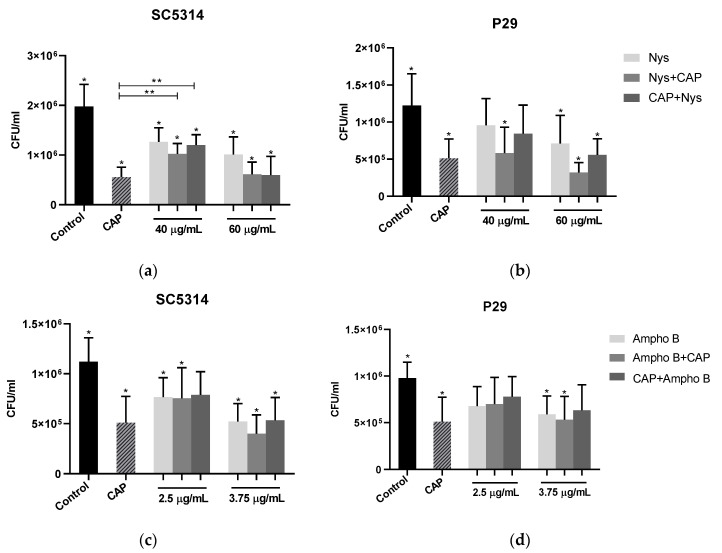
Values of CFU/mL of 24 h biofilms after associated treatments. (**a**,**b**) = isolated and associated treatments with Nystatin; (**c**,**d**) = isolated and associated with Amphotericin B. Control = no treatment; CAP = treatment with cold atmospheric plasma. Nys = treatment with nystatin; Ampho B = treatment with amphotericin B; Nys+CAP/Ampho B+CAP = previous treatment with nystatin or amphotericin B; CAP+Nys/CAP+Ampho B = previous treatment with CAP. (*) indicate significant differences (*p* < 0.05) between the groups and the control. (**) indicate significant differences (*p* < 0.05) between the isolated and associated treatments.

To assess the effects of the association between treatments, subinhibitory conditions were used. The CAP time was reduced to 2.5 min, while for polyene antifungals the concentrations were 75% (60 µg/mL for nystatin and 2.5 µg/mL for amphotericin B) and 50% (40 µg/mL for nystatin and 3.75 µg /mL for amphotericin B) in relation to the concentrations verified with antibiofilm effect (10 times MIC). The results were demonstrated by comparing all groups with the untreated control and between the association groups and the isolated treatments (CAP, Nys and Ampho B) (Figure 2).

In 24-h biofilms, there was a significant reduction (*p* < 0.05) in the number of viable cells after CAP exposure for both strains, compared to the control. For strain SC5314, only in the group treated with 2.5 µg/mL of amphotericin B with previous exposure to CAP (CAP+Ampho B), the reduction was not significant (*p* > 0.05) in relation to the control group. Furthermore, the results with the same strain showed that the isolated treatment with CAP was significantly more effective (*p* < 0.05), compared to associations with 40 µg/mL of nystatin.

For the P29 strain, in addition to the observations already mentioned regarding exposure to CAP, significant differences (*p* < 0.05) were observed with a reduction in the Nys+CAP group, with the previous treatment of 40 µg/mL of nystatin, and in all treatments using 60 µg/mL of the same antifungal, compared to the control. However, using amphotericin B, the significant treatments (*p* < 0.05) were in the Ampho B and Ampho B+CAP groups, using 3.75 µg/mL of the drug.

**Figure 3:**
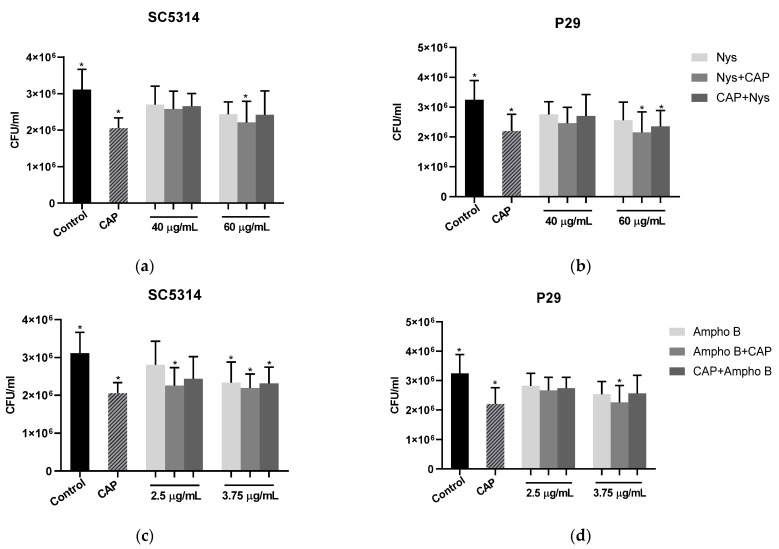
Values of CFU/mL of 48 h biofilms after associated treatments. (**a**,**b**) = isolated and associated treatments with Nystatin; (**c**,**d**) = isolated and associated with Amphotericin B. Control = no treatment; CAP = treatment with cold atmospheric plasma. Nys = treatment with nystatin; Ampho B = treatment with amphotericin B; Nys+CAP/Ampho B+CAP = previous treatment with nystatin or amphotericin B; CAP+Nys/CAP+Ampho B = previous treatment with CAP. (*) indicate significant differences (*p* < 0.05) between the groups and the control.

The effectiveness of the CAP treatment was also evidenced in the 48 h biofilms (Figure 3). There was a significant reduction (*p* < 0.05) in cells in the biofilms of both strains after treatment, compared to the untreated control. However, as verified in the 48-h biofilms, in the case of strain SC5314, using nystatin and amphotericin B, there was no significant difference (*p* > 0.05) between the isolated treatments and the associations of CAP and antifungal agents using 40 µg/mL for both strains.

Despite this, a significant reduction (*p* < 0.05) was observed with the association of Nys+CAP, using 60 µg/mL of nystatin previously, for both strains. With this same concentration of antifungal, a significant reduction (*p* < 0.05) in CFU/mL of the biofilms formed with the clinical isolate (P29) was also observed with the treatment after CAP exposition (CAP+Nys).

Regarding the control of 48 h biofilms, treatments with 3.75 µg/mL of amphotericin B prior to exposure to CAP (Ampho B+CAP) had a significant difference (*p* < 0.05) with a reduction for both strains. Still using this antifungal concentration, the reduction was evidenced in the isolated treatment and when the SC5314 strain biofilms were previously exposed to CAP (CAP+Ampho B). Furthermore, there was a significant decrease (*p* < 0.05) in cells in the biofilms of the same strain in the Ampho B+CAP group, using 2.5 µg/mL of amphotericin B compared to the control not treated.

## 3. Discussion

*Candida albicans* are fungal pathogens often associated with cases of mortality from systemic infection [27,28]. Biofilm formation, one of the virulence factors, stands out for its appearance on different surfaces [29] and for its greater infectious capacity [30] Biofilm infections of *C. albicans* can result in increased length of hospital stay, increased cost of antifungal therapy and risk of death [31].

Resistance to conventional antifungal agents has been observed in *C. albicans* biofilms [32,33] and may interfere in the development of therapies [31]. Considering that the biofilm is more resistant to antifungals when compared to planktonic cells, the treatments with antifungals used in this study, alone or in association with CAP, were carried out taking into account 10 times the initial MIC observed for nystatin and amphotericin B (80 and 5 µg/mL, respectively) after microdilution test.

Although all treatments alone reduced viable cells in *C. albicans* biofilms, exposure to CAP for 5 min had the most pronounced inhibitory effect compared to the other treatments. The effectiveness of CAP was also demonstrated in previous studies that reported the antibiofilm effect of cold plasma on *C. albicans* for the same exposure time with different plasma-generating equipment [24,34,35]. However, with the results verified in our study, it is possible to make a comparison with conventional drugs, demonstrating a possible alternative to antifungals.

Unlike conventional therapy, the occurrence of microbial resistance to cold plasma is unlikely due to its multiple forms of action and diversity of active agents [36,37,38]. Reactive species produced by CAP can induce oxidative damage to the biofilm during plasma exposure and, in this case, no specific interaction is required to induce the antifungal effect [39,40].

The microbial heterogeneity of the *Candida* biofilm, however, makes treatments difficult [41] and the combination of therapies with different mechanisms of action may be a more effective strategy. Combination therapies increase the treatment repertoire and can minimize an evolutionary resistance to antifungals due to different targets of action [42]. It is possible that synergistic interactions allow maintenance of the effect with dose reduction and potential toxicity [25].

Therefore, the present study evaluated the antibiofilm action of the association between CAP and antifungal agents with subinhibitory doses of each treatment. The time of exposure to CAP was 2.5 min, based on previous results that used the same parameters as this study, and demonstrated a reduction in biofilm from that period onwards, with low in vitro toxicity over 3 min [24]. Nystatin and amphotericin B concentrations were reduced to 75% (60 and 3.75 µg/mL, respectively) and 50% (40 and 2.5 µg/mL, respectively) of the values of 10 times MIC used in the treatments alone. Sardi et al. (2016) [43] demonstrated that nystatin concentrations up to 64 µg/mL have low toxicity in keratinocytes from the oral mucosa of humans, while Kagan et al. (2012) [44] found a reduction in fibroblast viability after exposure of cells to amphotericin B concentrations above 4 µg/mL. Therefore, dose reduction is an important factor for standardizing treatments in vivo.

Despite the use of subinhibitory doses, CAP exposure significantly reduced (*p* < 0.05) the CFU/mL of biofilms (24 and 48 h) formed by both strains, compared to the untreated control. The results show that there was no synergy, with no significant difference (*p* > 0.05) between the isolated treatments and the associations. Other authors verified the increased susceptibility of *C. albicans* cells to amphotericin B previously exposed to cold helium and oxygen plasma for 180 s. [27]. In this case, however, the CAP exposure parameters were different from our study, in addition to the concentration of amphotericin B (20 µg/mL) and the conditions of the fungal cells, which were in the planktonic form.

Studies have reported that exposure to CAP increases the permeability of fungal and bacterial cells [45,46,47], which would lead to greater susceptibility to drugs with the mechanism of action directed to the fungal membrane, such as polyene antifungals, which target molecules such as ergosterol, changing membrane permeability and leading to cell death [48]. However, despite this classic mechanism of action, there are reports that amphotericin B and other polyenes also act by inducing the accumulation of reactive oxygen species (ROS) [49]. The generation of reactive oxygen and nitrogen species (RONS) is also characteristic of CAP treatment, as is the production of atomic O and N and hydrogen peroxide, which may play a role in the inactivation of microorganisms [50]. Thus, the action of these different treatments could lead the cells to oxidative stress.

Different strategies have been developed by microorganisms such as *C. albicans* to respond to this stress to maintain redox homeostasis within the cell, which can ensure survival within the host [51]. An increase in the formation and metabolic activity of *C. albicans* biofilms was demonstrated in response to the stress induced by H_2_O_2_, with production of a thick exopolymer matrix to protect the cells, in addition to an increase in the production of proteinases [52].

Therefore, the absence of synergy, verified in our study, may indicate that the biofilm cells have previously suffered oxidative stress, in each association, stimulating protective activities and decreasing the antifungal action of the subsequent treatment. A similar protective behavior was suggested in a study with consecutive exposures of *C. albicans* to different concentrations of H_2_O_2_, with a reduced protective effect when the period between two exposures was greater than 120 min [53]. However, further investigations are needed to investigate possible resistance mechanisms related to the association of treatments.

The effects of combinations between antifungal treatments can also vary depending on the strains used. In associations with nystatin, it is possible to see different results for each clinical strain used [46], as well as in associations of amphotericin B and caspofungin, which may or may not have a synergistic effect, in different fungal strains of the same species [54].

Lastly, this was the first study to demonstrate the effects of antifungal and CAP combinations on *C. albicans* biofilms. Although synergy was not verified, it was possible to observe through comparisons between treatments, that CAP demonstrates more effective actions than conventional antifungal agents, being a viable alternative in future investigations for clinical applications.

## 4. Materials and Methods

### 4.1. Plasma Source

**Figure 4:**
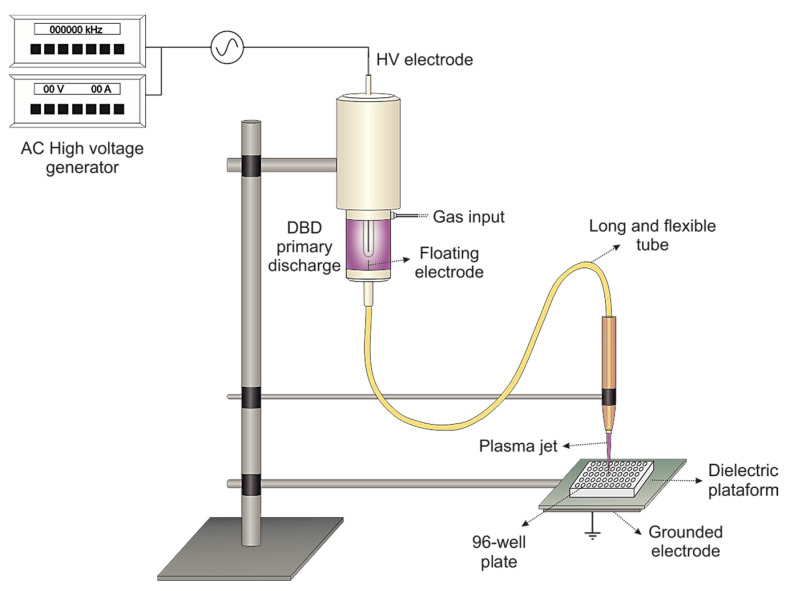
Experimental setup.

The cold atmospheric plasma (CAP) device (Figure 4) employed in this study was previously described in [24]. It basically consists of a syringe-like dielectric barrier discharge (DBD) reactor whose exit nozzle was connected to a 1.0-m-long plastic tube made of polyurethane with inner diameter of 2.5 mm. Inside this tube is placed a long floating copper wire (0.1-mm diameter) in such way that it slightly penetrates the DBD reactor and terminates few millimeters before the tube end. A 2-mm-diam Cu pin electrode, encapsulated in a closed-end quartz tube, is centered in the dielectric enclosure and serves as a powered electrode. It is connected to an AC power supply (Minipuls4, GBS Elektronik GmbH, Germany) that can generate AC voltage signal with amplitude up to 20.0 kV within the frequency range of 20 to 40 kHz. The working gas is introduced into the primary DBD reactor then it flows along the polyurethane tube and is ejected in the ambient air. When plasma is generated inside the DBD reactor, the floating wire becomes charged and a small plasma plume is ignited at the plastic tube distal end. This remote plasma jet can be manipulated by hand without risk of electric shock and this way easily directed to a target.

In this work, the resulting plasma jet was generated by an amplitude-modulated voltage signal that allowed a fine control over mean discharge power. In this mode, the AC voltage signal with frequency of 32.0 kHz and voltage amplitude of 13.0 kV was modulated forming consecutive bursts of high voltage oscillations (10 cycles) followed by around 1.2 ms voltage off period, thus resulting in a duty cycle of 22%. The system was fed with 2.0 slm (standard liter per minute) of 99.5% pure helium and the flow rate was controlled by a mass flow controller (N100 Horiba STEC, Osaka, Japan). In these conditions and using a distance of 15 mm between the tube tip and the broth surface, the calculated mean discharge power was around 1.0 W and the plume temperature did not superceed 40 °C. Thus, the above-mentioned distance was kept for all experiments.

### 4.2. Drug Stock Solutions

The stock solutions of antifungals nystatin (Sigma-Aldrich Co., St. Louis, MO, USA) and amphotericin B (Inlab, São Paulo, SP, Brazil) were prepared in dimethyl sulfoxide (DMSO, Synth, São Paulo, SP, Brazil) in concentrations 1.6 µg/mL, and kept in the dark at −20 °C until the use.

### 4.3. Strains and Growth Conditions

A reference strain of *Candida albicans* (SC5314) and a clinical isolate (P29) were used in this study. The strains were kept in Sabouraud dextrose broth with 20% glycerol at −80 °C. Fresh cultures were obtained by plating strains in SD agar and incubating at 37 °C for 24 h, under aerobiosis. In each experiment, standardized suspensions containing 10^6^ cells/mL in sterile saline solution (NaCl 0.9%) were prepared with the aid of a spectrophotometer. The clinical strain was isolated from prosthetic stomatitis lesions in previous study [28] with approval by the local ethics committee for human research (070/2006-PH/CEP).

### 4.4. Determination of Minimal Inhibitory Concentration (MIC)

Values of minimal inhibitory concentration (MIC) were determined by broth microdilution technique (CLSI, 2008) [55]. Briefly, microdilutions were prepared from stock solutions of nystatin and amphotericin B, in RPMI 1640 medium (Inlab, São Paulo, SP, Brazil) supplemented with 3-(N-morpholino) propane sulphonic acid (MOPS) (Inlab, São Paulo, SP, Brazil), in a 96-well plate, to obtain a concentration range from 32 to 0.062 µg/mL of each drug. Afterwards, 100 µL of fungal standardized suspensions (106 CFU/mL) prepared in RPMI were added in the wells and the plates at final concentration of 103 CFU/mL and were incubated at 37 °C for 24 h under aerobiosis. MIC values were determined as the lowest concentration without any visible growth (complete inhibition) for amphotericin B. For nystatin, MIC value was determined by the lowest concentration of drug that inhibited more than 50% of fungal growth in relation to control. Negative control was performed using just the culture medium, without treatment and fungal suspension. The experiments were performed in triplicate.

### 4.5. Biofilm Formation

Biofilms were formed in 96-well plates with *C. albicans* suspensions (10^6^ UFC/mL) incubated with RPMI 1640 medium supplemented with 2% glucose. Plates were incubated at 37 °C for 90 min under agitation (80 rpm) to adhesion phase. Afterwards, the wells were washed once with sterile physiological solution (NaCl 0.9%) to remove the non-adherent cells, and the culture medium was refreshed. Then, 200 μL of fresh RPMI supplemented with 2% glucose broth medium were added to each well and the plates were incubated at 37 °C for 24 h or 48 h.

### 4.6. Evaluation of Antibiofilm Effect of Isolated Treatments

At first, the biofilms were treated with the antifungals and CAP, separately, to confirm the effective antibiofilm conditions. The treatment with nystatin or amphotericin B was performed with exposition of biofilms (24 or 48 h) to drugs in determined concentrations (10 times MIC) for 5 min. In another moment, biofilms were exposed to CAP for 5 min, under the conditions previously reported [19]. After the treatments, biofilms were mechanically broken and, after a serial dilution, the suspensions were plated in SDA and incubated at 37 °C for 24 h. Biofilm viability was determined by counting CFU/mL. All the experiments were performed in triplicate in three different occasions (n = 9).

### 4.7. Determination of Antibiofilm Conditions with Treatments Association

After confirming the antibiofilm effect, the possible anti-biofilm effects of associations between antifungals and CAP were investigated. For these experiments, the period of exposure to CAP of 2.5 min was adopted in the same conditions evaluated before. This value corresponds to 50% of the exposure period with reported antibiofilm effect [20]. For the associations, the biofilms were exposed to nystatin or amphotericin B, both with 75% and 50% of 10 times MIC values used to evaluate antibiofilm effect, for 5 min, before or after CAP treatment. The dilution and incubation of cells, after the treatments, were performed as described before and the biofilm viability was also determined by counting CFU/mL. Isolated treatments with CAP and antifungals, in the same determined conditions, were also tested for comparative purposes.

### 4.8. Statistical Analysis

Data were analyzed using GraphPad Prism version 7.0 (GraphPad Software Inc., San Diego, CA, USA). The normality test was performed by Shapiro-Wilk normality test, and then the counted colonies were compared among the groups by One-way ANOVA and post hoc Tukey’s test. The level of significance was set at 5%.

## 5. Conclusions

In this study, the effects of the association between CAP and conventional polyene antifungals on *Candida albicans* biofilms was investigated. From the results reported in this work, it can be concluded that cold atmospheric pressure plasma was more effective against *C. albicans* biofilms than nystatin and amphotericin B. Furthermore, exposure to CAP was more efficient alone than in combination with conventional antifungal agents, demonstrating that the application of CAP may be a promising alternative for the treatment of oral candidiasis, in particular in cases refractory to conventional treatment.

## Data Availability

The data presented in this study are available on request from the corresponding author. Data will be soon available at http://hdl.handle.net/11449/202364.

## Sample Availability

Samples of the compounds are not available from the authors.
